# Predictors of packed red cell transfusion after isolated primary coronary artery bypass grafting – The experience of a single cardiac center: A prospective observational study

**DOI:** 10.1186/1749-8090-4-20

**Published:** 2009-05-07

**Authors:** Elsayed M Elmistekawy, Lee Errett, Hosam F Fawzy

**Affiliations:** 1 Division of Cardiovascular and Thoracic Surgery, St Michael's Hospital, University of Toronto, 30 Bond Street, Toronto, Ontario M5B 1W8, Canada

## Abstract

**Background:**

Preoperative patients' characteristics can predict the need for perioperative blood component transfusion in cardiac surgical operations. The aim of this prospective observational study is to identify perioperative patient characteristics predicting the need for allogeneic packed red blood cell (PRBC) transfusion in isolated primary coronary artery bypass grafting (CABG) operations.

**Patients and Methods:**

105 patients undergoing isolated, first-time CABG were reviewed for their preoperative variables and followed for intraoperative and postoperative data. Patients were 97 males and 8 females, with mean age 58.28 ± 10.97 years. Regression logistic analysis was used for identifying the strongest perioperative predictors of PRBC transfusion.

**Results:**

PRBC transfusion was used in 71 patients (67.6%); 35 patients (33.3%) needed > 2 units and 14 (13.3%) of these needed > 4 units. Univariate analysis identified female gender, age > 65 years, body weight ≤ 70 Kg, BSA ≤ 1.75 m^2^, BMI ≤ 25, preoperative hemoglobin ≤ 13 gm/dL, preoperative hematocrit ≤ 40%, serum creatinine > 100 μmol/L, Euro SCORE (standard/logistic) > 2, use of CPB, radial artery use, higher number of distal anastomoses, and postoperative chest tube drainage > 1000 mL as significant predictors. The strongest predictors using multivariate analysis were CPB use, hematocrit, body weight, and serum creatinine.

**Conclusion:**

The predictors of PRBC transfusion after primary isolated CABG are use of CPB, hematocrit ≤ 40%, weight ≤ 70 Kg, and serum creatinine > 100 μmol/L. This leads to better utilization of blood bank resources and cost-efficient targeted use of expensive blood conservation modalities.

## Introduction

Blood component transfusion has been an important part of coronary artery bypass graft surgery (CABG) since its inception [1]. Transfusion rates in cardiac surgery remain high despite major advances in perioperative blood conservation and institutions continue to vary significantly in their transfusion practices for CABG surgery [2-7]. The mean number of packed red blood cells (PRBCs) transfused in CABG ranges from 0 to 6.3 units per patient, and the frequency of transfusion ranges from 16% to 100% [1]. The National Blood Service for England issues approximately 2.2 million units of blood a year, of which 10% are used in cardiac surgical units [8,9]. Nearly 20% of all blood transfusions in the United States are associated with cardiac surgery [7,10].

In the early days of CABG, almost all patients received blood components or whole blood. However, despite current reductions in transfusion requirements for patients undergoing CABG, many patients continue to require transfusion [1]. Not all patients are at the same risk for transfusion requirement. Some patient variables can be used to predict the risk for perioperative transfusion [11].

Although often life-saving, blood transfusions are associated with significant risk to the patient and escalating costs to the blood bank system and hospital [12]. The risks associated with the use of allogeneic blood product transfusion include ABO/Rh incompatibility, sepsis, febrile reactions, immunosuppression, and viral transmission [10,13]. Transmission of hepatitis B and C and HIV by transfusion occurs in 1 in 300 000 cases, despite screening programs. Non-fatal but serious transfusion errors occur in 1 in 16 000 transfusions [14]. Blood transfusions have been linked to increased morbidity and mortality [15]. Homologous transfusions are immunosuppressive and associated with a higher risk of postoperative infection [16]. In addition, blood transfusion during or after coronary artery bypass operations were associated with increased length of intensive care unit and hospital stay [17]and long-term mortality [15,17,18]. Moreover, stored red cells undergo progressive structural and functional changes over time. In cardiac surgical patients, transfusion of red cells that had been stored for more than 2 weeks was associated with a significantly increased risk of postoperative complications as well as reduced short-term and long-term survival [19]. This is maybe explained by the fact that transfusion of old blood causing microvascular obstruction secondary to free cell fragments and strong proinflammatory effect [20,21]. Stored blood contains extracellular bioactive substances: Plasminogen Activator Inhibitor-1 (PAI-1) and IL-1beta which increase with the duration of blood storage [22]. Additionally, blood transfusion is a costly transplantation of tissue that may endanger the health of the recipient [23].

A number of pharmacologic and non-pharmacologic (mechanical) methods for reducing transfusion requirements are currently used [1]. Pharmacologic products to decrease blood use include 1-deamino-8-D-arginine vasopressin (DDAVP), tranexamic acid, epsilon-aminocaproic acid, and aprotinin [13]. The foremost mechanical methods of perioperative conservation of red blood cells are autologous blood donation, acute perioperative normovolemic hemodilution and intraoperative blood salvage [9,24,25]. The adoption of available blood conservation techniques, either alone or in combination, in patients undergoing cardiac surgery, could result in an estimated 75% reduction of unnecessary transfusions [7]. Both pharmacological and mechanical blood conservation methods have cost implications [9]. Preoperative identification of those at high risk for blood transfusion will allow for the use of cost-effective blood conservation modalities [11,26].

The aim of this prospective observational study is to identify preoperative (demographic, clinical, laboratory), intraoperative and postoperative patient characteristics predicting the need for allogeneic packed red blood cell (PRBC) transfusion in isolated, primary coronary artery bypass grafting in our local cardiac surgical service.

## Patients and methods

### Patient Population

After hospital's ethics committee approval, this prospective observational study included all patients who underwent first-time isolated coronary artery bypass grafting at North West Armed Forces Hospital, Tabuk, Saudi Arabia, during the period from May 2003 to April 2005 (n = 105). Patients were reviewed for their preoperative demographic, clinical (surrogate coronary artery disease severity and co morbidity) and laboratory variables and then followed till discharge to record their intraoperative data and postoperative outcomes.

All the patients included in the study were managed according to the hospital's current policies regarding preoperative preparation, intraoperative surgical and anesthetic management, and postoperative care.

### Surgical Technique

All the patients included in this study were operated upon by one surgical team. Operations were done either on-pump or off-pump. Routinely, left internal mammary artery was used as in-situ pedicled graft for the left anterior descending coronary artery. Arterial conduits (right internal mammary artery and radial artery conduit) were used whenever possible according to patient's characteristics and saphenous vein grafts were used for completion of revascularization.

### Preoperative Preparation

All cardiac medications were continued till the day of surgery except for antiplatelet drugs which were stopped 5 days prior to surgery in elective patients except when the expected risk of acute coronary events was higher than risk of bleeding. Risk stratification of patients was done by means of Euro SCORE (standard/logistic). In the current study, emergency CABG was defined as surgical intervention indicated within 72 hours from coronary angiographic diagnosis or acute coronary event to prevent death or major morbidities.

### Anesthetic Technique

Anesthetic technique was standardized in all patients. Patients were premedicated with midazolam 7.5 mg orally the night before surgery and morphine 10 mg intramuscularly 30 minutes before sending the patient to theater. Anesthesia was induced with midazolam 0.05 – 0.1 mg/kg, fentanyl 5 – 10 μg/kg, and propofol 1 – 2 mg/kg. Pancuronium 0.15 mg/kg was used to facilitate endotracheal intubation and mechanical ventilation. Patients were mechanically ventilated to keep EtCO_2 _between 30 – 35 mmHg. Anesthesia was maintained with sevoflurane 1 – 1.5 MAC and N_2_O 50% in oxygen. Fentanyl and pancuronium supplements were administered as required.

### Conventional On-pump CABG

On-pump CABG operations were done via median sternotomy. After Heparin was given in a dose of 300 IU/kg to reach target activated clotting time ≥ 480 seconds, cardiopulmonary bypass (CPB) was established. Moderate hypothermia (28 – 32°C) was used throughout CPB. The prime volume of the circuit consisted of 1,500 mL of Plasmalyte-A and 25 gm of mannitol. Blood was added to keep hematocrit (Hct) on the CPB around 20 – 22%. Cold antegrade multidose blood cardioplegia (Modified St. Thomas cardioplegic solution) was used to induce and maintain diastolic cardiac arrest. Mean arterial blood pressure was kept at 50 – 70 mmHg. After the conclusion of all anastomoses and termination of CPB, intravenous protamine was used to neutralize circulating heparin.

### Off- pump CABG

Off-pump patients were operated upon through median sternotomy. After harvesting conduits and making pericardial sling, heparin 150 IU/kg was administered, and supplemental doses were added, as needed, to maintain activated clotting time (ACT) between 200 – 250 seconds. Different Maneuvers have been used to help exposure of the target vessels like Medtronic Octopus III, Starfish Repositioner, deep pericardial stitch and Intracoronary shunts. After completing of the anastomoses, the total dose of heparin was reversed with protamine (1:1 ratio).

### ICU Management

Patients were transferred to ICU intubated and were mechanically ventilated until they were ready for weaning. Monitoring, sedation, analgesia, and inotropic and vasoactive drug administration were managed according to ICU protocols.

### Blood Conservation Techniques

For on-pump operations, moderate hemodilution was used during CPB. All blood remaining in the circuit of CPB was retransfused to the patient at the end of the procedure. In off-pump cases, cell saver (Brat 2 Autologous Blood Recovery System, Cobe Cardiovascular, Inc., Arvada, CO, USA) was routinely used except for cases that needed only one anastomosis (LIMA-to-LAD grafting). No prophylactic antifibrinolytic medications were used during the entire period of the study.

### Transfusion Guidelines

Blood was transfused during CPB if hematocrit value was less than 20% and/or hemoglobin was less than 7 gm. Postoperatively, blood transfusion was started if hemoglobin was less than 8 gm and/or hematocrit was less than 25% according to the departmental protocol unless the patient was showing signs of hemodynamic instability or ongoing bleeding. Usually two units of packed RBCs were used in a single transfusion setting.

### Study Design

The study endpoint was packed red blood cell (PRBC) transfusion. Data were reported both categorically (i.e. whether PRBCs were used or not) and quantitatively (number of units transfused). Clinical events such as reexploration for bleeding, morbidity and mortality were reported. Multivariate logistic regression models were built to assess the predictors of packed red blood cell transfusion. The transfusion of fresh frozen plasma and platelets was not included in this analysis.

### Statistical Methods

SPSS for Windows, Release 7.5.1, statistical package (SPSS, Inc., Chicago, IL, USA) was used for statistical analysis. Patients' preoperative, intraoperative and postoperative variables and PRBC transfusion data were presented as mean ± SD or number (%) as appropriate. Student's t-test or Mann-Whitney U test were used to compare numerical variables and Pearson chi-square test, Yates' correction, or Fisher's exact test were used to compare ordinal variables between transfused and non-transfused patient population. Then, logistic regression analysis was performed to select the best predictors of PRBC transfusion. Univariate analysis was done first to detect significant predictors, followed by multivariate regression analysis models. Continuous variables were analyzed twice, first as continuous and then as dichotomous variables. Cut points were derived from mean, median, high or low normal, or previously defined values. A P value < 0.05 was considered significant.

## Results

This prospective observational study comprised 105 patients presenting for primary isolated coronary artery bypass grafting. Patient population consisted of 97 males (92.4%) and 8 females (7.6%), with mean age 58.28 ± 10.97 (range, 31 – 90) years. CABG operations were either elective (82 patients, 78.1%) or emergency (23 patients, 21.9%). On-pump operations were 48 (45.7%) and the remaining 57 (54.3%) were done off-pump. The number of distal anastomoses ranged from 1 – 5, with a mean of 3.02 ± 0.88. In patients who underwent on-pump CABG, mean CPB time was 99.53 ± 24.13 minutes and mean aortic cross-clamp time was 62.85 ± 21.06 minutes.

**Figure 1:**
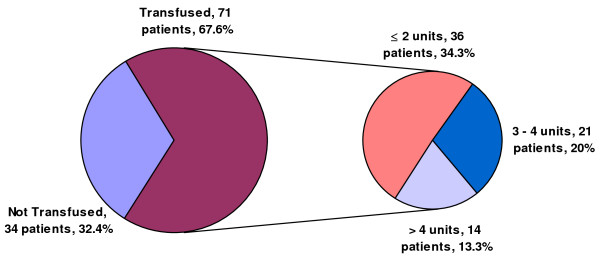
**Packed red blood cell transfusion data**. Of the 105 patients included, packed red blood cell (PRBC) transfusion was utilized in 71 patients (67.6%). Thirty-five patients (33.3%) received more than 2 units of PRBC; of these, 14 patients (13.3%) received more than 4 units.

Of the 105 patients included, packed red blood cell (PRBC) transfusion was utilized in 71 patients (67.6%). Thirty-five patients (33.3%) received more than 2 units of PRBCs; of these, 14 patients (13.3%) received more than 4 units. A total of 227 units of PRBCs were used, with a mean of 2.16 ± 2.25 units per patient. The mean number PRBC units received was 1.21 ± 1.58 in off-pump patients and 3.27 ± 2.45 in on-pump patients (P = 0.026) (figure 1).

All the eight female patients included in the study (8/8, 100%) received PRBC transfusion compared with 63 male patients (63/97, 64.9%). Patients who received PRBC transfusions had significantly older age and lower body weight, body surface area (BSA), body mass index (BMI), and preoperative hemoglobin (Hb) and hematocrit (Hct). Transfused and non-transfused patient populations didn't differ significantly as regards height, platelet count, Prothrombin time (PT), Partial Thromboplastin time (PTT), serum creatinine, and serum albumin. Associated co morbidity percentages were comparable in transfused and non-transfused groups, including current smoking, diabetes mellitus, hypertension, cerebrovascular disease, chronic obstructive pulmonary disease, and peripheral vascular disease.

Also, transfused patients had significantly higher Euro SCORE (logistic and standard) compared with non-transfused patients. Left ventricular ejection fraction (LVEF) was comparable in transfused and non-transfused patients. Number of patients who were receiving heparin in the preoperative period and those in whom antiplatelet therapy was continued in the last 5 preoperative days were comparable in transfused and non-transfused populations. Surgical intervention was elective in 76.1% (54/71) and emergency in 23.9% (17/71) of transfused patients while in non-transfused patients surgery was elective in 82.4% (28/34) and emergency in 17.6% (6/34) (P = 0.47). CPB was used in 57.7% (41/71) of transfused patients and in only 20.6% (7/34) of non-transfused patients (P = 0.0003). Patients who received perioperative PRBC transfusions had more distal anastomoses than patients who were not transfused (3.14 ± 0.88 vs. 2.76 ± 0.82, respectively, P = 0.04). Use of variable types of conduits for grafting was comparable in transfused and non-transfused patients, except for the radial artery (used in 74.6% of transfused and 50% of non-transfused patients, P = 0.01). In on-pump patients, CPB time and aortic cross clamp time showed no significant differences between transfused and non-transfusion patients.

Postoperatively, patients who received PRBC transfusions had significantly higher chest tube drainage than those who were not transfused (1005 ± 727 vs. 632 ± 207 mL). PRBC transfusion was significantly associated with prolonged intubation time and hospital stay, but not Cardiac Surgical Unit (CSU) stay. Perioperative myocardial infarction, neurologic dysfunction, pneumonia, wound infection, and mediastinitis didn't show a significant association with transfusion. In-hospital mortality was recorded in 4 patients (3.8%), all of them received PRBC transfusion (P > 0.05).

Using derived cut points, transfused patient population, compared to non-transfused, included significantly more patients > 65 years old (33.8% vs. 14.7%), ≤ 70 kg body weight (60.6% vs. 23.5%), ≤ 1.75 m^2 ^BSA (60.6% vs. 35.3%), ≤ 25 BMI (43.7% vs. 11.8%), ≤ 13 gm/dL Hb (43.7% vs. 23.5%), ≤ 40% Hct (59.2% vs. 29.4%), > 100 μmol/L creatinine (38% vs. 11.8%), > 2 Euro Score (standard, 57.7% vs. 32.4%; logistic, 53.5% vs. 32.4%), ≥ 1000 mL postoperative chest tube drainage (35.2% vs. 5.9%).

Table 1. Is showing pre-operative patients' characteristics, operative surgical data and post-operative outcome data. Univariate analysis showed that significant preoperative predictors of perioperative PRBC transfusion were: female gender, age > 65 years, body weight ≤ 70 Kg, BSA ≤ 1.75 m^2^, BMI ≤ 25, preoperative hemoglobin ≤ 13 gm/L, hematocrit ≤ 40, serum creatinine > 100 μmol/L, and Euro SCORE (logistic/standard) > 2. Significant intraoperative and postoperative predictors were use of cardiopulmonary bypass, higher number of distal anastomoses, radial artery use, and postoperative chest tube drainage > 1000 mL.

**Table 1:** Preoperative patients' characteristics, operative surgical data, and post-operative outcome data.

**Variable**		**Total**	**Transfusion**	**No Transfusion**	**P Value**
Gender	Male	97 (92.4%)	63 (88.7%)	34 (100%)	0.1002
	Female	8 (7.6%)	8 (11.3%)	0 (0%)	

Age (years)	Mean ± SD	58.28 ± 10.97	60.46 ± 10.69	53.73 ± 10.36	0.0028
	≤ 65 years	76 (72.4%)	47 (66.2%)	29 (85.3%)	0.0405
	> 65 years	29 (27.6%)	24 (33.8%)	5 (14.7%)	

Weight (Kg)	Mean ± SD	72.67 ± 14.81	69.76 ± 12.05	78.74 ± 18.10	0.0032
	≤ 70	51 (48.6%)	43 (60.6%)	8 (23.5%)	0.0003
	> 70	54 (51.4%)	28 (39.4%)	26 (76.5%)	

B.S.A. (Kg/m^2^)	Mean ± SD	1.75 ± 0.15	1.72 ± 0.15	1.79 ± 0.11	0.0249
	≤ 1.75	55 (52.4%)	43 (60.6%)	12 (35.3%)	0.0152
	> 1.75	50 (47.6%)	28 (32.4%)	22 (64.7%)	

B.M.I.	Mean ± SD	27.07 ± 4.18	26.39 ± 4.52	28.49 ± 2.96	0.0155
	≤ 25	35 (33.3%)	31 (43.7%)	4 (11.8%)	0.0011
	> 25	70 (66.7%)	40 (56.3%)	30 (88.2%)	

Hb (gm/dL)	Mean ± SD	13.59 ± 1.52	13.31 ± 1.59	14.18 ± 1.18	0.0055
	≤ 13	39 (37.1%)	31 (43.7%)	8 (23.5%)	0.0457
	> 13	66 (62.9%)	40 (56.3%)	26 (76.5%)	

Hct (%)	Mean ± SD	39.18 ± 4.00	38.42 ± 4.07	40.99 ± 3.21	0.0062
	≤ 40	52 (49.5%)	42 (59.2%)	10 (29.4%)	0.0043
	> 40	53 (50.5%)	29 (60.8%)	24 (70.6%)	

Creatinine (μmol/L)	Mean ± SD	106.79 ± 83.58	106.63 ± 53.82	107.12 ± 125.98	0.9780
	≤ 100	74 (70.5%)	44 (62%)	30 (88.2%)	0.0057
	> 100	31 (29.5%)	27 (38%)	4 (11.8%)	

Type of surgery	On-pump	48 (45.7%)	41 (57.7%)	7 (20.6%)	0.0003
	Off-pump	57 (54.3%)	30 (42.3%)	27 (79.4%)	

Use of RA		70/105 (66.7%)	53/71 (74.6%)	17/34 (50%)	0.0121

Number of distal anastmoses		3.02 ± 0.88	3.14 ± 0.88	2.76 ± 0.82	0.0390

CPB time (min)		99.53 ± 24.13	104.85 ± 24.88	98.60 ± 24.22	0.4731

Ao × time (min)		62.85 ± 21.06	64.74 ± 19.87	51.85 ± 25.86	0.1347

Intubation time (hours)		29.24 ± 78.06	37.11 ± 94.28	13.03 ± 5.680	0.03674

CSU stay (days)		7.31 ± 15.40	7.54 ± 16.25	6.85 ± 13.66	0.8227

Hospital stay (days)		9.19 ± 6.82	9.90 ± 8.08	7.67 ± 1.77	0.0294

Postoperative blood loss					
Mean ± SD		883.59 ± 632	1005 ± 727	632 ± 207	0.0043
≤ 1000 mL		78 (74.3%)	46 (64.8%)	32 (94.1%)	0.0012
> 1000 mL		27 (25.7%)	25 (35.2%)	2 (5.9%)	

Data are presented as mean ± SD or No. (%).

B.S.A.: Body Service Area. B.M.I.: Body Mass Index.

Hb: Hemoglobin. Hct: Hematocrit.

RA: Radial Artery.

CPB time: Cardiopulmonary Bypass time.

Ao × time: Aortic cross clamp time. CSU: Cardiac Surgery Unit.

By multivariate regression analysis, the strongest predictors of blood transfusion were the use of CPB (P = 0.0001), preoperative hematocrit ≤ 40 (P = 0.002), body weight ≤ 70 kg (P = 0.01), and serum creatinine > 100 (P = 0.01) (Table 2).

**Table 2:** Transfusion risk and regression logistic analysis of perioperative variables associated with blood transfusion.

**Variable**	**Transfusion vs. No Transfusion**		**Transfusion Risk**		**Univariate Analysis**		**Multivariate Analysis**
		
	**t-test**	**X**^**2**^	**OR**	**CI (95%)**	**Numerical**	**Categorical**	
Gender	-	0.1002	-	-	-	0.014	

Age	0.0028	0.0405	2.962	1.017 – 8.625	0.0050	0.0335	

B.M.I.	0.0155	0.0011	5.813	1.852 – 18.244	0.0212	0.0026	

B.S.A.	0.0249	0.0152	2.815	1.204 – 6.582	0.0288	0.0169	

Hemoglobin	0.0055	0.0457	2.519	1.003 – 6.325	0.0077	0.0493	

Euro SCORE (standard)	0.0052	0.0149	2.858	1.210 – 6.747	0.0082	0.0166	

Euro SCORE (logistic)	0.0200	0.0571	2.408	1.022 – 5.670	0.0284	0.0444	

No. of Distal anastomoses	0.0390	-	-	-	0.0431	-	

Radial artery use	-	0.0121	2.944	1.247 – 6.951	-	0.0137	

Postoperative bleeding	0.0043	0.0012	8.696	1.922 – 39.335	0.0086	0.0050	

Use of CPB	-	0.0003	5.271	2.028 – 13.704	-	0.0007	0.0001

Hematocrit	0.0062	0.0090	3.476	1.447 – 8.350	0.0013	0.0039	0.0021

Weight	0.0032	0.0003	4.991	1.980 – 12.579	0.0093	0.0007	0.0105

S. creatinine	0.9780	0.0005	4.602	1.460 – 14.506	0.9778	0.0092	0.0142

B.M.I: Body Mass index.

B.S.A: Body surface area.

CPB: Cardiopulmonary Bypass.

## Discussion

The use of allogeneic blood transfusion after coronary artery surgery is still high despite published transfusion guidelines and costly blood conservation strategies [27,28]. Readily available patient variables can predict patients at risk for transfusion [29]. The classification of coronary artery bypass graft patients on the basis of attributes known preoperatively and by conduits used yields subsets of patients with distinctly different transfusion requirements and in-hospital outcomes [30].

Prediction models based on preoperative variables may facilitate blood component management and improve the efficiency of ordering blood before operations for patients undergoing CABG surgeries in order to assist blood banks in improving responsiveness to clinical needs [1,31].

Two patterns are usually prevailing for preoperative blood component stocking and preparation in cardiac surgical patients. One practice is the cross-matching of a large number (4 or more) of PRBC units that are usually – at least in part – not utilized, leading to wasting blood bank efforts and resources, including time, space, and reagents. The other is cross-matching a limited number of units (usually 2 units of PRBCs) and typing an extra 2 units to be cross-matched at need. This may lead to a blood bank emergency when a patient is in urgent need for blood component transfusion, putting a considerable stress and time load on the blood bank team. A model to predict patients at risk of requiring more than the standard number of blood components would alleviate plenty of blood bank stress, save time and resources, and allow better utilization of available space and blood resources [1].

Blood conservation has become one of the most important issues in cardiac surgery [29,32]. Some of blood conservation strategies are cost-efficient and simple to utilize and can be employed in nearly all cardiac surgical patients without adding further risk to the patient or effort to the operating room team, including non-hemic prime of cardiopulmonary bypass machine, salvage of blood from surgical field using cardiotomy suction, hemodilution during CPB, retransfusion of all contents of oxygenator at the end of CPB, and use of ultrafiltration and modified ultrafiltration during and after CPB. Use of other modalities, such as antifibrinolytic therapy, preoperative autologous blood donation, use of cell saving devices, and auto transfusion of shed mediastinal chest tube drainage is still limited owing to doubts about their effectiveness and inappropriateness for use in many patients [8,33-36]. In addition, the routine use of such expensive and sophisticated techniques in all cardiac surgical patients causes higher cost exceeding the benefit obtained. So, these strategies should be utilized more critically [37].

The use of predictors of allogeneic PRBC use in CABG patients allows for preoperative risk stratification and may allow for more rational resource allocation of costly blood conservation strategies [28]. Identifying high-risk patients for transfusion would alter perioperative patient management and allow the employment of a multimodality approach to blood conservation resulting in a lower transfusion rate at a reasonable cost-efficiency [38].

In our study, 67.6% (71 patients) of all patients undergoing primary isolated CABG operations either on pump or off-pump received PRBC transfusion, 33.3% of the total number (35 patients) needed more than 2 units, and only 13.3% (14 patients) needed more than 4 units of PRBCs. The mean number of PRBC units received was 2.16 ± 2.25 unit per patient.

The strongest predictors of PRBC use in our study were the use of CPB (P = 0.0001), preoperative hematocrit ≤ 40 (P = 0.0021), body weight ≤ 70 kg (P = 0.0105), serum creatinine > 100 μmol/L (P = 0.0142). Other predictors included female gender, age > 65 years, BSA ≤ 1.75 m^2^, BMI ≤ 25, hemoglobin ≤ 13 gm/dL, Euro SCORE > 2, radial artery use, and higher number of distal anastomoses (a cut point could not be derived).

Current smoking; associated comorbidities (including diabetes mellitus, hypertension, cerebrovascular disease, COPD and peripheral vascular disease); preoperative platelet count, PT, PTT, and serum albumin; left ventricular EF; type of intervention (elective/emergency); type of conduits used (apart from radial artery); and duration of CPB and aortic cross-clamping were not significant predictors for PRBC transfusion in our patients. Also, preoperative use of heparin or continuation of antiplatelet treatment beyond the last 5 preoperative days didn't prove as significant predictors of PRBC transfusion.

Clinical studies conducted to identify perioperative risk factors and predictors of blood component use in cardiac surgical procedures varied widely regarding study designs, surgical procedure characteristics, study period, and study end points.

In a Japanese study, *Isomatsu et al. (2001) *[32] studied 89 patients undergoing isolated CABG surgery over 2 years, from 1997 to 1999, to determine preoperative predictors of the need for blood transfusions during CABG surgery. Of these, 66 patients (74%) received transfusion during hospitalization. Independent predictors were emergency surgery, lower hematocrit, older age, and presence of peripheral vascular disease. Optimal cutoff of hematocrit was 39% and age 64 years.

*Karkouti et al. (2001) *[26] studied all patients undergoing elective first-time CABG surgery prospectively from January 1997 to September 1998 in Ontario, Canada. Transfusion rate was 29.4 percent. Predictors included preoperative hemoglobin, weight, age, and sex.

In a retrospective analysis of 400 consecutive patients undergoing CABG, including emergency and re-operations, *Litmathe et al. (2003) *[11] found that 132 patients (33%) received PRBC transfusion during hospitalization. On average, 2.2 ± 0.68 units of red cell concentrate were transfused per patient. Predictive parameters were age > 70 years, preoperative hemoglobin < 11 gm/dL, reoperation, and ejection fraction < 0.35. The authors could not find any significantly increased red cell concentrate transfusion in female cases, insulin dependent diabetes mellitus, or impaired renal function.

*Scott et al. (2003)*[39] studied impact of CPB, hematocrit, gender, age, and body weight on blood use in 1235 consecutive patients undergoing primary CABG over a period of 2 years under on-pump or off-pump technique. PRBC transfusion was used in 72.5% of on-pump patients compared with 45.7% of off-pump patients. A lower percentage of males (52.6%) than females (79.4%) received transfusion. Use of CPB, preoperative hematocrit < 35%, female gender, increasing age (≥ 65 years), and decreased body weight (≤ 83 Kg) were significant predictors of transfusion. The strongest predictors of PRBC transfusion were preoperative Hct < 35% and use of CPB.

*Arora et al*., in *2004 *[28], studied 3,046 consecutive, isolated CABG patients over 3 years to identify independent predictors of allogeneic blood product transfusion. Allogeneic blood was used in 23% of all patients and 16.9% of isolated, elective, first-time CABG cases. Independent predictors of blood product usage were preoperative hemoglobin 12 gm/dL or less, emergent operation, renal failure, female sex, age 70 years or older, left ventricular ejection fraction 0.40 or less, redo procedure, and low body surface area. The authors validated this model on 2,117 consecutive isolated CABG patients.

*Al-Shammari et al. (2005) *[40] reviewed the medical records of 159 consecutive primary CABG patients retrospectively to determine the perioperative factors associated with intraoperative blood transfusion. Overall, 128 (80.5%) patients received blood product transfusion intraoperatively, 113 (70.5%) of them received PRBCs and the remaining received fresh frozen plasma and platelets. Moreover, 23 patients (12.6%) received more than two PRBC units intraoperatively. Totally, 159 patients consumed 342 units of PRBCs at an average of 2.1 units per patient. Significant factors associated with intraoperative RBC transfusion were: age > 60 years, female gender, preoperative hemoglobin < 12 gm/dL, and 3 or more coronary bypass grafts constructed.

*McDonald and McMillan*, in *2005 *[41], utilized the product of BSA (m^2^) and preoperative hemoglobin (gm/L) as an index for intraoperative blood transfusion, the Transfusion Predictor Product (TPP). For patients with TPP less than 211.7 units, 46% received blood transfusion intraoperatively. At a TPP greater than 211.7 units, 6% of patients had intraoperative blood transfusion. They suggested that patients with a TPP > 211.7 do not require routine cross-matching of blood.

The role of increased postoperative chest tube drainage should be considered as the cause of lowering postoperative hemoglobin/hematocrit to the level necessitating PRBC transfusion according to the transfusion guidelines employed and not as a predictor.

Preoperative coagulation parameters (platelet count, PT, PTT) couldn't prove to be significant predictors for PRBC use in our study. This can be explained by the very small number of patients in our study with subnormal platelet count or prolonged PT and PTT due to our center's adherence to a strict policy regarding preoperative coagulation status. In elective cases, antiplatelets are stopped for at least 5 days preoperatively except if strongly indicated, highest PT and PTT allowed for CABG surgery is 15 seconds and 44 seconds, respectively, and the lowest platelet count accepted is 150 × 10^3^/L. In emergency cases, patients not fulfilling these criteria are usually postponed for few hours up to 72 hours for control of their coagulation parameters unless earlier/immediate surgical intervention is indicated. This accounts to the narrow range reported in this study for PT (9 – 15 seconds) and PTT (28 – 46 seconds), thus ameliorating the effect of these otherwise important parameters as predictors of bleeding/transfusion.

Although use of CPB and number of distal anastomoses are intraoperative events, rather than preoperative; yet, in the vast majority of cases, both are usually planned from preoperative parameters (including patient's general condition and demographic variables, left ventricular function, and coronary angiography data about number and sites of diseased coronary vessels). Changes from off-pump to on-pump and omitting anastomoses based on intraoperative findings and/or events are usually kept within a narrow range. So, use of CPB and number of distal anastomoses still maintain their validity as preoperative predictors for blood preparation and use of blood conservation modalities.

Radial artery use was found statistically significant as a predictor for PRBC use in the perioperative setting. This could not be explained in light of previous researches or literature. We couldn't determine if this was a mere coincidental association or due to the association of radial artery use with other verified predictors for transfusion, e.g. number of distal anastomoses and use of CPB.

We chose body weight (and not BSA or BMI) for analysis in most models owing to the fact that univariate analysis and multivariate analysis proved that weight is a stronger predictor for PRBC transfusion than BSA and BMI. Also, Hct was used in most multivariate analysis models instead of Hb as it was a stronger predictor for transfusion than Hb by univariate analysis.

The strongest predictor of PRBC use in our study was the use of CPB. Of 48 patients in whom CPB was used, 41 (85.4%) received PRBC transfusion compared with 30 patients (52.6%) of 57 off-pump patients. Off-pump CABG eliminates the risks of cardiopulmonary bypass and the systemic inflammatory response it elicits [42].

A retrospective review of 744 patients undergoing multivessel CABG either on-pump (n = 609) or off-pump (n = 135) was carried out by *Kshettry et al. (2000) *[43]. Postoperative blood loss and use of blood transfusions were significantly less in patients operated upon off-pump.

Three other studies were in agreement with our results. *Nuttal et al. (2003) *[44] retrospectively studied charts of 200 adult patients who underwent CABG either on-pump or off-pump. Although heparin was not reversed at the end of OPCAB patients, OPCAB surgery was associated with an overall reduction in allogeneic transfusion requirements.

In a large series, *Frankel et al. (2005) *[42] compared 3646 off-pump CABG patients with a contemporaneous control group of 5197 on-pump CABG patients. Off-pump CABG was associated with a lower need for single and multiple unit postoperative blood transfusions compared to on-pump CABG patients.

On the contrary, *Gerola et al. (2004) *[45], in a multicenter randomized study on 160 selected low-risk patients undergoing CABG on-pump (80 patients) or off-pump (80 patients), did not find any statistical difference in blood component use between on-pump and off-pump patients; 43.7% of on-pump patients and 43% of off-pump patients received blood component transfusion. Number of blood units used in on-pump patients was 2.9 ± 1.8 unit per patient and 2.2 ± 1.3 unit per patient in off-pump patients.

In our study, out of 620 units of PRBCs cross-matched for 105 patients undergoing first-time isolated CABG (4 units for patients) according to our hospital's policy, only 192 units were transfused (30.9%) and another 35 units were cross-matched and transfused in the 14 patients who received > 4 units of PRBCs. This reflects the necessity of developing a blood reservation policy considering patients individually based on their predicted transfusion risks. Such a policy would have saved > 50% of blood bank's efforts and resources.

Among the limitations of this study is the small number of patients included due to the small size of our center's target population. Another limitation is restricting the study to PRBC transfusion predictors only. There is a strong need in our center for determining the predictors of fresh frozen plasma and platelet transfusion in cardiac surgical procedures. The cost-efficiency of application of a blood conservation strategy targeting patients at risk of transfusion needs verification through prospective clinical studies.

## Conclusion

Need for PRBC transfusion in isolated, first-time CABG patients can be predicted preoperatively. The strongest predictors are use of CPB, hematocrit ≤ 40%, body weight ≤ 70 Kg, and serum creatinine > 100 μmol/L. Female gender, older age, higher Euro SCORE, and number of distal anastomoses are also significant predictors. Ability to identify patients at risk of PRBC transfusion would save blood bank efforts and resources and allow the employment of a targeted blood conservation policy in CABG patients.

## Competing interests

The authors declare that they have no competing interests.

## Authors' contributions

EME conceived of the study, participated in its design and carried out the coordination, collecting, analyzing the data, performing the statistical analysis and writing the manuscript. LE participated in reviewing the manuscript. HFF conceived of the study, participated in its design, writing, reviewing and submitting the manuscript. All authors read and approved the final manuscript.
